# Quality of non-expert citizen science data collected for habitat type conservation status assessment in Natura 2000 protected areas

**DOI:** 10.1038/s41598-017-09316-9

**Published:** 2017-08-21

**Authors:** A. S. Kallimanis, M. Panitsa, P. Dimopoulos

**Affiliations:** 1 Department of Ecology, Aristotle University, Thessaloniki, Greece; 2 Department of Environmental and Natural Resources Management, University of Patras, Agrinio, Greece; 3 Faculty of Biology, Division of Plant Biology, University of Patras, Patras, Greece

## Abstract

EU biodiversity conservation policy is based on the Habitats Directive (92/43/EC), which aims that habitat types and species of Community interest should reach ‘favourable conservation status’. To this end, Member States are obliged to perform periodic assessment of species and habitat conservation status through biodiversity monitoring, which, in almost all cases, was performed by experts implementing standardized field protocols. Here, we examine the quality of data collected in the field by non-experts (citizen scientists) for the conservation status assessment of habitat types, and specifically for the criteria ‘typical species’, ‘specific structures and functions’, and ‘pressures and threats’. This task is complicated and demands different types of field data. We visited two Natura 2000 sites and investigated four habitat types (two in each site) with non-experts and compared their data to the data collected by experts for accuracy, completeness and spatial arrangement. The majority of the non-expert data were accurate (i.e. non-experts recorded information they observed in the field), but they were incomplete (i.e. non-experts detected less information than the experts). Also, non-experts chose their sampling locations closer to the edge of the habitat, i.e. in more marginal conditions and thus in potentially more degraded conditions, than experts.

## Introduction

The biodiversity conservation policy of EU is primarily based on the Natura 2000 network of protected areas. Natura 2000 was established in the framework of two Directives – the Birds (79/409/EEC; as updated in 2009/147/EU) and the Habitats (92/43/EEC) Directives^1, 2^. Today, Natura 2000 is the largest conservation network of protected areas in the world with more than 26,500 sites covering more than 17% of the total land area of the EU and 6% of its territorial waters, across all 28 Member States of EU^3^.

The aim of the EU nature conservation policy and of the Natura 2000 network is that habitats and species of community interest should reach and maintain ‘favourable conservation status’ so that their long-term persistence will be secured^4^. Article 1 of the Habitats Directive defines ‘conservation status’ as the sum of the influences on habitats or species that affect their long-term distribution, structure and functions, area/abundance, or future prospects. Species and habitats of community interest are defined as the species and habitats listed in the annexes of the Habitats Directive. National governments are responsible to establish appropriate site management practices to ensure that the condition of the natural habitats and species are maintained and/or restored to favourable conservation status. Article 17 requires Member States to report every six years about the progress towards the implementation of this goal, i.e. on how species and habitat types of community interest maintained and/or were restored to favourable conservation status^5^.

Thus, for the successful implementation of the EU nature conservation policy, the conservation status of habitat types and species of community interest needs to be assessed. Here we examine the assessment for habitat types. According to the Habitats Directive conservation status could be characterized as Favourable (FV), Unfavourable - Inadequate (U1), or Unfavourable - Bad (U2), using the criteria: (i) area, (ii) range, (iii) structures and functions, and (iv) future prospects and trends. The conservation status of a habitat type is favourable when the habitats natural range and the area of occupancy are stable or increasing, and the specific structures and functions which are necessary for its long-term maintenance are present and are likely to continue to exist for the foreseeable future (Directive 92/43/EC Article 1e).

There are guidelines on what criteria to use. However, until now, the methodology on how to measure these criteria is not standardized^5^. And indeed different Member States assessed conservation status using different methodologies to quantify the four criteria and different thresholds to classify the scores to conservation status classes. Two of these criteria (range and area) could be estimated only at the national level, using different methods (e.g. mapping, modelling) and different data sources (e.g. remote sensing data and/or other geographic information)^5, 6^. But, GIS and remote sensing can get us only so far and a critical part of the assessment requires field work^7, 8^. In the case of habitat types, the most crucial part of the conservation status assessment that relies on fieldwork is the assessment of the structures and functions (i.e. information on the specific structures and functions of each habitat type including the presence, abundance and vitality of typical species) and the future prospects (which relies on the pressures and threats operating on the habitat types and how they are likely to affect the habitat in the foreseeable future)^4^.

The first critical step was to define the specific structures and functions of each habitat type, which should include characteristics of the habitat that indicate healthy ecosystem and correspond to the ecological requirements of the different habitat types. These characteristics may include natural features (like diversity of dominant species age classes, or rich understory plant diversity) or lack of indications of anthropogenic degradation (e.g. no apparent signs of logging, or planted species). An important part of this criterion specifically mentioned in the Habitats Directive is the typical species. These are species that characterize the habitat type and may belong to one of the following categories: (a) common species (often including the dominant species) of the habitat type, (b) species representing biogeographic differentiation. Therefore, these indicators of quality were specified for each habitat type and biogeographic region separately by each EU Member State. We used the protocols developed and applied in Greece for the conservation status assessment of habitat types. Finally, to assess the future prospects of the habitat type in a location, the pressures and threats operating on it need to be considered. To this end, the diversity and intensity of pressures and threats, both natural processes (e.g. diseases, biotic invasions, climate change) and human activities (e.g. agriculture, forestry, industry), operating in an area are recorded^9^. The list of pressures and threats is common for all EU countries.

The monitoring schemes that were used to produce the Article 17 reports on the conservation status assessments of habitat types were based almost exclusively on field data collected by experts. For species of community interest there were some monitoring schemes utilizing data collected from volunteers or citizen scientists^10, 11^. But no such collaboration was evident for habitat type conservation status assessment. One major impediment in the use of volunteers for habitat type conservation status assessment is the complexity of the necessary information. One critical part of the information needed is the presence and cover/abundance of typical species. But habitat type conservation status assessment also requires information on the quality of the habitat, as indicated by specific structures and functions, and some basic estimation of the pressures and threats to the habitat to allow for predictions in the foreseeable future. To our knowledge no citizen science scheme attempted for the same people to collect such complex and different levels of information (species, habitat structures, pressures and threats) for a sample area simultaneously. So far, citizen science programs focused on the collection of simple information (such as the presence or abundance of one or few species)^12–14^. There are examples of citizen science projects for monitoring from the more familiar taxa like birds^15, 16^ or butterflies^17^ to less noticeable like saproxylic beetles^18^. And several citizen science programs focus on alien invasive species^19–21^. The first challenge was to see if non-experts could collect all types of information necessary for the conservation status assessment of habitat types.

There are a range of citizen science monitoring approaches depending on the contribution of the scientists and that of the public. On the one end of the spectrum, there are contributory projects, i.e. projects designed by scientists where the public primarily contributes data. There are collaborative projects i.e. projects designed by scientists but with substantive input from the public, e.g. regarding the aims of the study or the analysis of the data. And, there are also co-designed projects (i.e. projects designed by scientists and members of the public in close collaboration and where citizens participate in all stages of the scientific process and not only the collection of the data). In the present study, given the very specific needs of monitoring the habitats conservation status in accordance with the Habitats Directive, we examined the case where the public contributes only data to a scientific study designed by experts.

The second challenge was to assess the quality of the data collected by non-experts. Data quality assessment is a fundamental question regarding data collected by citizen scientists^22–25^. Most of the existing citizen science monitoring programs refer to simple data like the presence of a species or a group of species^25, 26^ and the general consensus is that the citizen science data are useable and highly valued by decision makers and scientists^27^. Their quality is considered accurate and comparable to experts when recording simple observations like the presence of species, but their accuracy decreases as more complex data are collected^28^.

To this end, on two different Greek Natura 2000 areas (GR2530001: Summits of Mountain Killini and Flambouritsa gorge (Mt Ziria) and GR2320001: Kalogria lagoon, Strofilia forest and Lamia’s marsh), we deployed teams of experts and non-experts to collect data on the conservation status assessment of two habitat types in each area (four habitat types in total). Data were collected for sample plots of 100 m^2^. In Strofilia, we studied habitat types 2120 (mobile dunes forming the seaward cordon of dune systems of the coasts) and 2270 (inner coastal dunes, stabilized part of the coastal sandy dune system). In Mt Ziria, we studied habitat types 5210 (evergreen sclerophyllous scrub organized around arborescent junipers) and 9560 (Endemic forests with *Juniperus* spp). The non-experts team in each case was larger (60 and 70 persons) than the team of experts (2 and 3 experts).

The study has two aims. The first aim is to test if non-experts can collect the necessary data for conservation status assessment, which is a complicated task that demands data on three types of indicators (i. structures and functions, ii. typical species, iii. pressures and threats). The second aim is to evaluate the quality of the collected data by comparison with the data collected by experts for the same habitat type and in the same location. For the second aim, the quality assessment will be based on three criteria: (1) accuracy, (2) completeness, and (3) spatial arrangement of sampling sites.

## Results

### Specific structures and functions

**Figure 1:**
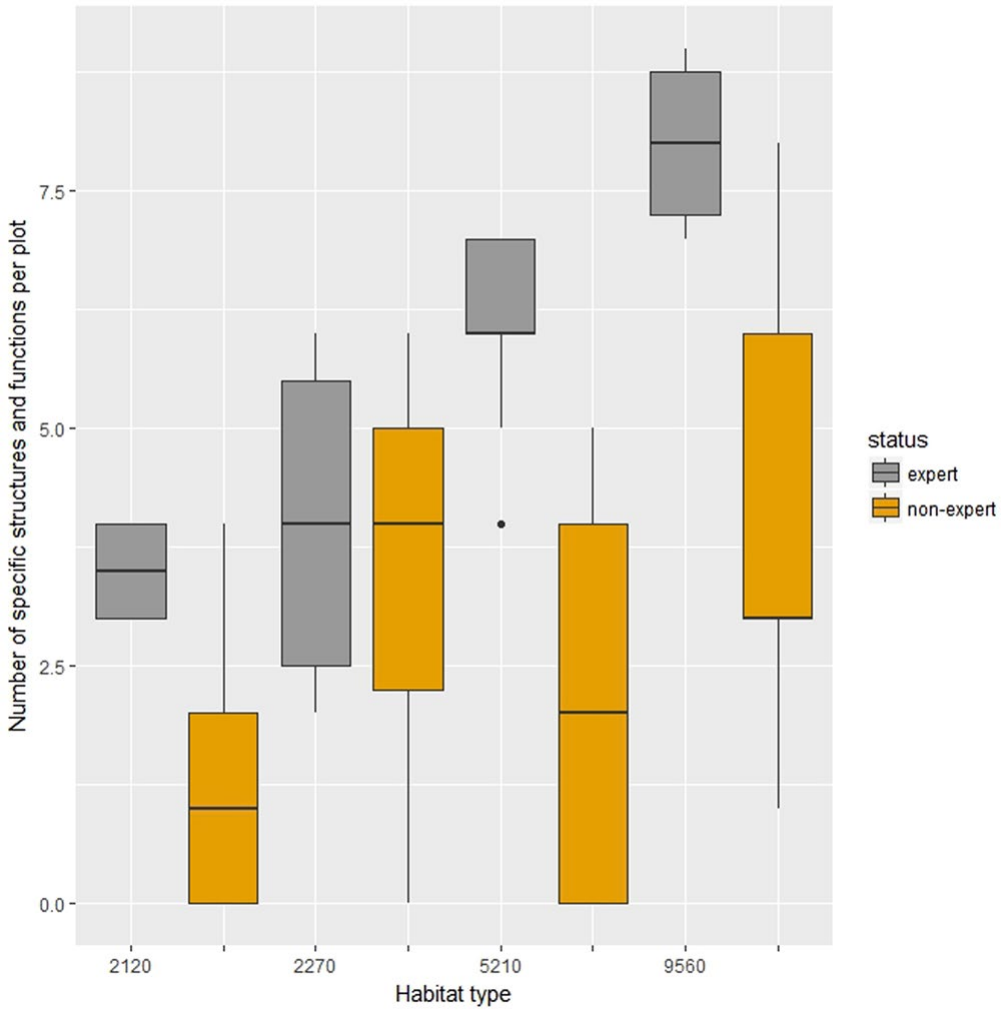
Boxplots of the number of specific structure and functions recorded per 100 m^2^ sampling area for the different habitat types. Habitat types 2120 (mobile dunes forming the seaward cordon of dune systems of the coasts) and 2270 (inner coastal dunes, stabilized part of the coastal sandy dune system) were investigated in Strofilia site (Natura 2000 site code GR2320001); habitat types 5210 (evergreen sclerophyllous scrub organized around arborescent junipers) and 9560 (Endemic forests with *Juniperus* spp) were investigated in Mt Ziria (Natura 2000 site code GR2530001). With grey colour is the records made by experts and with orange colour is the records made by non-experts. The line represents the median, while the boxes represent the 50% interval, and the whiskers the 95% intervals, outliers are presented as dots.

The non-experts systematically underestimated the number of specific structures and functions per plot compared to the experts (Fig. 1). Permutational ANOVA showed that the experts recorded per sampling area significantly more structures and functions than non-experts (p < 0.001). If we analyze the data for the different habitat types as well, we see that the habitat types did not differ significantly in the number of structures and functions recorded per plot (p > 0.9). When analyzing the data for each habitat type separately, in all cases the difference between experts and non-experts was significant (p < 0.01), with the exception of habitat type 2270 (inner coastal dunes) where difference was not significant (p > 0.2).

However, if we accumulate the reports of experts and non-experts, the picture changes. In the case of Mt Ziria and habitat type 9560 non-experts cumulatively recorded 10 structures and functions in the 31 plots examined while the experts only 9 structures and functions in the 6 plots examined. The structure and function reported only by the non-experts was reported in only one plot of the non-experts and it was the proportion of the soil covered by litter to be greater than 20%. For the habitat type 5210, the non-experts reported 7 structures and functions in 41 plots, while the experts 8 in 15 plots. The experts reported 3 structures and functions that none of the non-experts reported (indicators regarding the conditions of the herb layer, which was not apparent during the non-experts visit). On the other hand, the non-experts reported 2 structures and functions that the experts did not report. More specifically, in 13 plots the non-experts reported the presence of grazing, and in 6 plots they reported that the understory was dominated by low shrubby species. Similarly for the Strofilia protected area, non-experts reported structure and functions that the experts did not report. In the case of habitat type 2120, in 5 plots non experts reported sighting of dunes landwards of the *Agropyretum juncei*, which the experts did not report.

And when using this data to assess the conservation status according to the above mentioned criteria, the picture is different. The plots examined by the experts within the area covered by habitat type 9560 in Mt Ziria site are all classified as Favourable (FV), while only 10 of the plots examined by non-experts are FV, 14 are Unfavourable-Inadequate (U1) and 7 are Unfavourable-Bad (U2). In the case of the habitat type 5210 of the 15 plots examined by experts 13 are classified as FV and 2 as U1; while for the plots examined by non-experts 7 are classified as FV, 12 as U1 and 2 as U2. Similarly for the habitat type 2120 among the plots examined by experts 2 were assessed as FV and 2 as U1, while among the plots examined by non-experts 1 was classified as FV, 28 as U1 and 31 as U2. For the habitat type 2270 in the Strofilia site, among the plots examined by experts 2 were classified as FV, 2 as U1, and 2 as U2, while among the plots examined by non-experts, 36 were FV, 11 were U1 and 11 were U2. So for the criterion specific structures and functions, the non-experts data indicated worse conditions than the experts’ data.

### Typical species

**Figure 2:**
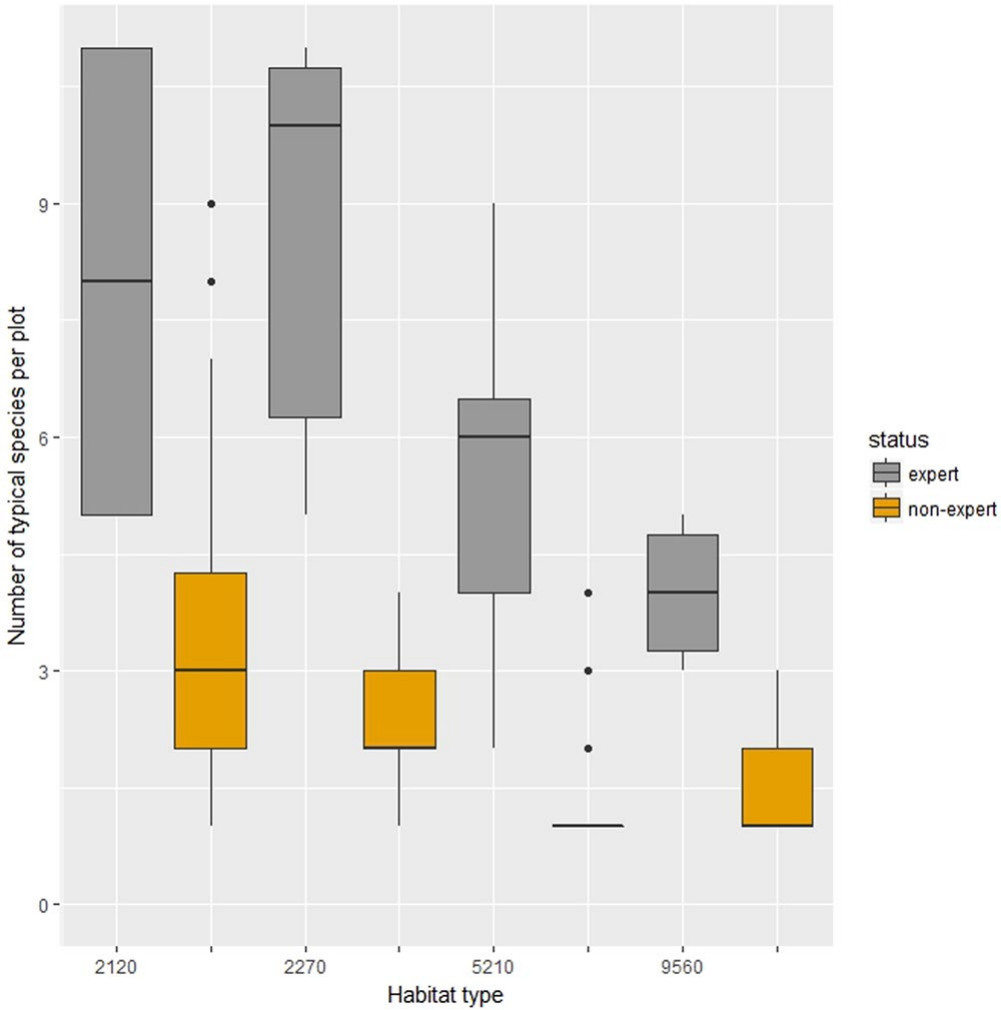
Boxplots of the number of typical species recorded per 100 m^2 ^ sampling area for the different habitat types. With grey colour is the records made by experts and with orange colour is the records made by non-experts. Habitat codes, box and whiskers as in Fig. 1.

For the typical species there are similar patterns of non-experts identifying systematically fewer species per sample plot than experts (Fig. 2). This was verified by permutational ANOVA where the difference of typical species per plot among experts and non-experts differed significantly (p < 0.001). The different habitat types did not differ significantly in the number of typical species reported (p > 0.9). Analyzing the data for each habitat type separately, it was verified that experts reported more typical species per plot than non-experts (with p < 0.001 in all habitat types).

Cumulatively, the image is different in the two study sites. In the Mt Ziria site, the experts recognized far more species than the non-experts, which might be explained by the date of the non-experts visit (late autumn). As far as woody species are concerned, the non-experts identified most species reliably. For example in the habitat type 5210, most of the non-experts identified the dominant shrub species (*Juniperus oxycedrus*) but did not report any species of the herbaceous layer. This might reflect the timing of the field visit to Mt Ziria (late autumn when most herbs are not apparent). But in the case of the Strofilia site, where experts and non-experts visited the site at the same time, the cumulative results of the two groups are reverse. There is a group of species reported by both groups (11 species in habitat 2120 and 6 in habitat 2270) and there are few species reported only by experts (far less than in the Mt Ziria site) but there are also species recorded by non-experts that were not recorded by experts (10 species in habitat 2120 and 6 in habitat 2270). The difference may be due to the difference in sampling effort (with 60 non-experts vs 3 experts). Still there are two examples of species recorded by a single non-expert each, that the experts consider unlikely to have been observed in the Strofilia area. The remaining species were reported by many non-experts each, and are species known to occur in the region.

### Pressures and threats

**Figure 3:**
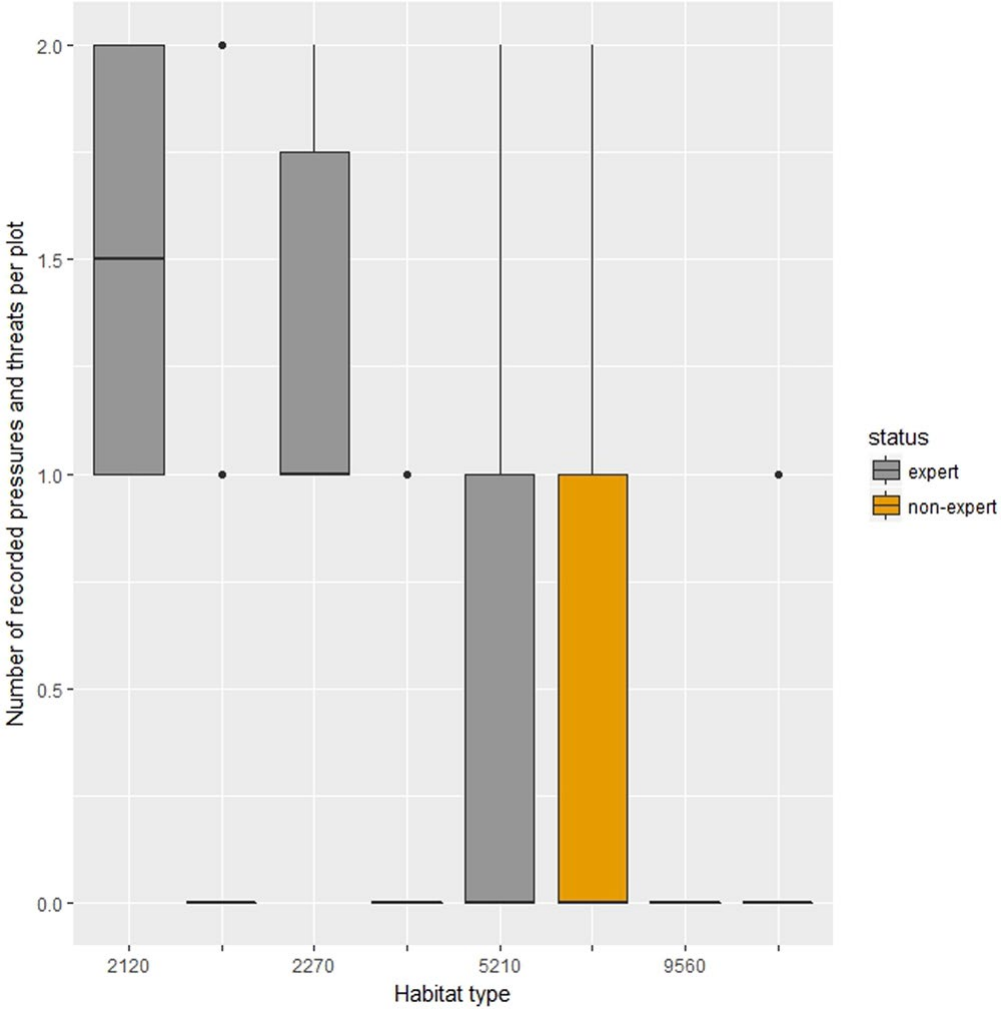
Boxplots of the number of pressures and threats recorded per sampling area for the different habitat types. With grey colour is the records made by experts and with orange colour is the records made by non-experts. Habitat codes, box and whiskers as in Fig. 1.

Regarding the pressures and threats, experts and non-experts, assessing the same sites, report few and low intensity activities. In almost all plots, non-experts reported no pressures and threats observed, while the experts approximately in half of the plots report no pressure or threat and in the remaining plots they reported one and rarely two pressures (Fig. 3). Still the difference is significant according to permutational ANOVA (p < 0.001). However, this is mainly due to habitat types 2120 and 2270 (permutation ANOVA p < 0.001); since in habitat types 5210 and 9560 the differences were not significant (permutational ANOVA p > 0.6).

Cumulatively, the non-experts reported more pressures and threats than the experts, in both sites. In the case of the habitat type 5210 both groups reported the grazing and mowing activity taking place at low to medium intensity. However, non-experts also recorded several human activities, like outdoor sports, roads, buildings and structures. These activities are indeed taking place in the area, but are at the edge of the habitat type and not the core, so their reporting might indicate that the non-experts chose to sample areas that are closer to the road network at the edge of the habitat type, while the experts preferred sites further away from human presence to the core area of the habitat type.

And in order to use this data to assess the conservation status according to the criteria of future trend and future prospects of the sampled area where the habitat is present, in all habitat types (both for Strofilia and Mt Ziria), both expert and non-expert assessment indicates FV status for all plots.

## Discussion

### Ability of citizen scientists to collect data needed for conservation status assessment

The citizen science approach has been applied in many biological monitoring programs where the volunteers are asked to report sightings of specific species or groups of species, and some rudimentary environmental information. Here we tested this approach to a more complicated task, gathering information for the conservation status assessment of habitat types, and more specifically information on the habitats structures and functions, typical species and pressures and threats. The first outcome is that it worked and data were collected. But the question with this, as with any dataset collected by non-experts, is the quality and the shortcomings of this data in comparison to data collected by experts^29, 30^.

The task is demanding. A team of two experts typically needs about half an hour in the field to collect all the relevant data from a single sample plot. In our case, the non-experts needed between an hour and a half and two hours for the same task. So the citizen science approach could be applied to such complicated tasks, but the non-experts require considerably more time and at least some preliminary training before going to the field to carry out the task.

### Quality assessment of citizen scientist data

The quality of such data is always the critical step for appreciating and utilizing such data^25^. In our case, the main conclusion is that the majority of the non-experts did record information they observed in the field, but they observed less information than the experts. Another possible shortcoming in the data is that the non-experts chose their sampling locations closer to the edge of the habitat, i.e. in more marginal conditions and thus in potentially more degraded conditions. Per sampling plot, they reported less structures and functions, less typical species and equal or less pressures and threats, compared to the experts that visited the same area. This decreased detection ability of non-experts compared to experts, especially for low density species has also been reported in other assessments of citizen science data quality^27, 31^. But there is a possible improvement, in the case of non-experts we should also consider the wisdom of the crowds rather than the individual, so we should compare not only the records per sampling area but also the cumulative records, and in that case the increased sampling effort of many more non-experts lead to comparable results with the few observations collected by experts.

So, as far as the accuracy of the reported observations is concerned, our analysis shows that most records are reliable, as far as presences are concerned. Experts recorded something similar in the region, or know that this is likely in the study area. The few examples that the non-experts reported something that the experts did not consider likely are rare and were single records out of the group. On the other hand, the observations of the experts were also recorded by many of the non-experts. So, when analyzing the data cumulatively, the outliers, i.e. records of something that only few observers reported should be marked and handled with caution. If not possible to verify these unique records in the field, the analyst would be advised to downweight or even omit these observations from further analysis.

We had several novel observations from non-experts compared to experts. More specifically, several non-experts record pressures and threats related to human activities (e.g. roads, buildings, outdoor sports) that the experts did not record. We were able to verify that these activities do take place in the study areas, but they affected the examined habitat type only at its edge. These records indicate that the non-experts preferred sampling locations near the edge of the habitat type (close to roads and paths) that the experts avoided since they sampled the more typical conditions of the habitat type and not its margins. So in future practice, we propose that the non-experts should receive more training on the selection of appropriate sampling locations. However, it should be acknowledged that the experts focus primarily on the ecologically optimum conditions of the area, and systematically avoid edge habitats; thus scientists may sometimes block out the social dimensions of the ecological sampling underway.

As far as typical species are concerned, the non-experts did indeed identify most woody species reliably. However, the non-experts failed to locate many species that the experts recorded in these same areas. And for the Mt Ziria site, this might be attributable to the inappropriate date of sampling. In late autumn early winter, many herbaceous plants are dormant and thus not observed in the field. But this was also observed (even though it was less pronounced) in the case of the Strofilia site, where experts and non-experts visited the site only a couple of days apart, and still the non-experts systematically recorded fewer species per plot than the experts. So the completeness of the data recorded by the non-experts is relatively low. This aspect seems to be affected by the life-form of the organism recorded (woody plant species are more systematically detected than herbaceous plant species) but it seems not to be affected by the conditions of the location since they are observed in both coastal and mountainous locations in both open landscapes and more shrub dominated formations. Also it should be pointed out that typical species used in such assessments are common species and so they are easy to find, but perhaps the limited a priori knowledge made their identification harder.

### Concluding remarks

But could we use this data to assess the conservation status of the examined habitat types? The answer is to use them with caution. At the individual level (analysis at the scale of sample plot), while most of the experts records led to a classification of FV (Favourable status); many of the non-experts classified the conditions as FV, but the majority of them classified it as U1 (Unfavourable-Inadequate). Few records were even classified as U2 (Unfavourable-Bad). This outcome is partly because non-experts examined sample plots close to the edge of the habitat type, where conditions were more disturbed and less typical, and partly because the non-experts as individuals observed less than the experts. However, if we accumulate the data, remove outliers (records that are unique and not repeated by other observers) the non-experts as a group record almost everything that the experts recorded and even some things that the experts did not record. Thus, we suggest that such approaches are useful and reliable when viewed at the level of the group and not the individual plot.

## Materials and Methods

### Conservation status assessment protocols for habitat types

Even though the conservation status assessment of habitat types is an EU legal obligation (article 17, Habitats Directive) and the basic criteria to be used (area, range, specific structures and functions and future prospects) are common for all Member States, the methodological approach for this estimation is not uniform across Member States. Typically, the criteria of area and range are estimated at the national level, by means of habitat mapping based either on field surveys or modelling or a combination of both. However, the criteria of specific structures and functions and future prospects rely exclusively on extensive field work. So far, this field work was undertaken by experts. There are examples of citizens that offered data for the conservation status assessment of plant and animal species^11^ but not for the habitat types monitoring.

In Greece, the assessment, at the plot level, was based on variables-criteria expressing the completeness and status of (i) structure and functions (including typical species), as well as (ii) the future trend and (iii) the future status of the habitat’s structures and functions and relied on sampling protocols that were applied in the field by experts. Τhe status and completeness of structure & functions (including typical species) play the major role in the assessment at the plot level. Moreover, in the assessment process, the actual status, the future trend and the future status of the habitat area of occupancy per Site of Community Importance (Natura 2000 protected area) was assessed for its sufficiency to allow for the habitat’s mid-term conservation. The protocol was established as part of the third national report (for the period 2007–2012) on the conservation status assessment for the purposes of Article 17 of the Habitats Directive. The protocol consisted of four sections: general information on the sampling area, structure and functions, typical species and pressures and threats.

### Specific structures and functions

The general information included coordinates, altitude, reference to previous sample (if it existed) and general information on the geomorphology of the area (slope, aspect, soil conditions etc.) and the vegetation coverage. This first part was common for all habitat types. The other parts were specific for each habitat type, and they even were differentiated geographically if the habitat type was known to have biogeographic variation. According to Art 1(e) of the Habitat Directive, for the conservation status of a habitat to be favourable, “the specific structure and functions which are necessary for its long-term maintenance exist and are likely to continue to exist for the foreseeable future”. The structure of a habitat type is the physical organization of the habitat type (e.g. the horizontal and vertical components of a woodland). While functions reflect the ecological processes operating in the area (e.g. regeneration of the dominant species, decomposition of dead wood). Often assessing functions directly is not a trivial task and thus we may rely on surrogate indicators, e.g. saproxylic beetles as indicators of decomposition of dead wood. Specific structures and functions included characteristics of the habitat type that indicate healthy ecosystem. These characteristics are very different among habitat types, and their selection was a task performed by experts at the Greek national level. These characteristics may include natural features (like diversity of dominant species age classes, or rich understory plant diversity) or lack of indications of anthropogenic degradation (like logging, or planted species). These characteristics formed a predefined checklist that the assessor in the field had to estimate as true or false. The precise lists used in the four habitat types could be found in the Supplementary material.

It is not considered necessary that all structures and functions should be present in all areas, but the more of these features recorded in a site the better the condition of the habitat type regarding its structure. More specifically, if more than 50% of the structures and functions are present in a site then for this criterion the conservation status is Favourable (FV). If the proportion is between 25% and 50% then the status is Unfavourable – Inadequate (U1). If the proportion is less than 25% then the status is Unfavourable - Bad (U2). Τhe use of algorithms reduce the effect of subjective expert judgment.

### Typical species

The presence and condition of the typical species of each habitat is an important criterion mentioned in the Habitats Directive 92/43/EC. But no satisfactory definition of typical species is proposed. Typical species may include all species groups, for example plants, lichens, mosses as well as all animal groups. Different Member States used different approaches. For example, in France typical species was considered as indicative species of ecosystem’s functions, thus focusing on species functional traits^32^. In the Greek protocols, typical species were identified based on their representativity of the plant community that characterizes the habitat type. This includes species on which the identification of a habitat type is found (e.g*. Pinus nigra* for priority habitat type 9530 - Mediterranean pine forests with endemic black pines). This includes species that are always present in the habitat type. This does not mean that the species distribution is restricted to only this habitat type, but it also includes species that are characteristic of the habitat type but not restricted to it, so some species may appear as typical in different habitat types. The definition of typical species was a laborious task since even within the Greek territory different habitat types displayed distinct biogeographic differentiation and different species were considered typical in different regions^33, 34^. Thus, once a predefined list of typical species was specified per habitat type, then the abundance and vitality of these species was recorded in sample plots taken in the field. The species are characterized by different weights based on how representative of the habitat type they are and also based on their vitality and abundance. The more typical species present and the greater their vitality, the status is more favourable.

The assessment of the conservation degree of the habitat types, to a large extent is based on quantified criteria provided to the evaluators as “checklist”. The abundance of the typical species in the sample plot was reported as “abundant”, “frequent”, “occasional” and “rare”.

### Pressures and threats

Finally, the human pressures and threats applied in the sampling area were recorded. For this task there is a rather exhaustive classification of pressures and threats to habitat types, both natural and human, compiled by the European Environment Agency-European Topic Centre on Biological Diversity) and was applied by all Member States (http:biodiversity.eionet.europa.eu/article17/reference portal).

In the context of Article 17 reporting, pressures are past and present impacts that threaten the long-term viability of a species or of a habitat and act now and/or during the current 6-year reporting period. Threats are future/foreseeable impacts that affect the long-term viability of a habitat and are expected to act in the future after the current reporting period (future two reporting periods, i.e. 12 years following the end of the current reporting period). If an activity is already taking place and will continue in the foreseeable future then it is classified as a pressure and a threat. For each pressure and threat there should also be an estimate of its intensity (as low, medium or high).

The greater the number and intensity of pressures and threats in an area the more negative the future prospects of the habitat type. If no pressures and threats are recorded (or only one of low or medium intensity) then for the criterion future prospects and trend the status is Favourable (FV). Otherwise, if up to three pressures or threats of low or medium intensity are recorded then the status is Unfavourable Inadequate (U1). In all other cases (i.e. at least one activity of high intensity or more than three of moderate intensity) then the status is Unfavourable Bad (U2).

The protocols used in the present study are in the Supplementary material.

### Data collection

These protocols are designed so as to collect the necessary scientific information aiming to the habitats conservation status assessment. Although this type of information is typically collected by experts, we want these protocols to be used and completed to the field not only by experts, but also by the wider public. More specifically we tested this approach using distinct groups of citizens scientists (two groups were volunteers non-experts University students of ages 20–22), and of experts employed in the surveillance, monitoring and drafting of the Greek National Conservation Status Assessment Report. The choice of environmental science students between the ages of 20 and 22 as citizen scientists may not represent a random sample of volunteers with interest in citizen science. Citizen scientists may be characterized by a more diverse range of ages and include people with more free time, such as retirees which significant life experience. However, in Greece volunteer movements are still scarce, and many citizen science projects in Greece is related to education and typically include young people. Still a potential limitation of our findings might be the restricted range of ages, since age is a factor that may affect the quality of citizen science data^35^.

We deployed these groups in two Natura 2000 sites: Summits of Mountain Killini (Ziria) and Flambouritsa gorge (code: GR2530001, here called Mt Ziria site), and Kalogrias Lamias wetands and Strofylia forest (code GR 2320001, here called Strofilia site).

In the high elevation zone of the Mt Ziria site, two teams (2 experts and 70 non-experts) evaluated habitat types: 9560 (Endemic forests with *Juniperus* spp), and 5210 (Arborescent matorral with *Juniperus* spp). In this case each habitat assessment protocol (i.e. each sample plot) was examined by a group of 2 or 3 non-experts.

Habitat type 9560 is a priority habitat type that is characterized as Mediterranean medium to high altitude open-canopy forest formations dominated by arborescent *Juniperus* spp., mainly on calcareous substrata. Many different sub-types are included in this habitat type with different geographical distribution along Europe. The habitat type with *Juniperus excelsa* and *J*. *foetidissima* occurs in the Balkans; while the type with *J. drupacea* occurs in the southern Peloponnese. Greece is highly responsible within EU for the conservation of *Juniperus excelsa* and *J. foetidissima* woodlands and exclusively for the Peloponnesian *J. drupacea* stands.

Habitat type 5210 is characterized as Mediterranean and sub-Mediterranean evergreen sclerophyllous scrub organized around arborescent junipers. The following sub-types are included: Arborescent matorral dominated by *Juniperus oxycedrus* s.l.; Arborescent matorral dominated by *Juniperus phoenicea* s.l.; Arborescent matorrals of Greece, Anatolia and the Near East, dominated by *Juniperus excelsa* or *J. foetidissima*; Mediterranean formations dominated by *Juniperus communis*; Arborescent matorrals dominated by *J. drupacea* distributed to the Peloponnese and Asia Minor.

In the coastal area of the Strofilia site, two teams (3 experts and 60 non-experts) evaluated two habitat types: 2120 (Shifting dunes along the shoreline with *Ammophila arenaria*), and 2270 (Wooded dunes with *Pinus pinea* and/or *Pinus pinaster*). In this case, each habitat assessment protocol (i.e. each sample plot) was examined by an individual non-expert.

Habitat type 2120 is mobile dunes forming the seaward cordon of dune systems of the coasts; psammophilous, perennial, hemicryptophytic/geophytic vegetation, colonizing the inner, higher coastal dunes (defined as “shifting” or “white” dunes), dominated by *Ammophila arenaria*.

Habitat type 2270 is a priority habitat type and is inner coastal dunes (stabilized part of the coastal sandy dune system), from Mediterranean to Temperate (Submediterranean) macrobioclimate, colonized (or sometimes forested) by Mediterranean thermophilous pine species (*Pinus halepensis, P. pinea, P. pinaster)*.

The effort the experts applied in collecting the data is representative of the sampling effort that was used for assessing the conservation status of each habitat type in each Natura 2000 site for the purposes of the Greek national Article 17. Typically 2 to 3 experts worked in each site collecting at least 3 plots per habitat type. The number of experts employed in our study is far lower than the number of non-experts (60 to 70 non-experts for 2–3 experts). This reflects the relative availability of the two groups (there are far more non-experts than experts) and the advantage of citizen science as a crowd sourcing application, i.e. the use of a large group of individuals of varying knowledge, experience and training to voluntarily undertake a task typically performed by experts. Also it should be pointed out that this large difference in number of experts vs volunteers did not impede other studies from assessing the quality of citizen science data. For instance, Galloway *et al*.^36^ compared tree diameter/crown assessments of a group of 607 students to that of 8 proffesionals^36^. Similarly, Fuccilo *et al*.^37^ compared phenological data collected by 28 volunteers with the records of one expert^37^. In the study of Finn *et al*.^38^ 3 experts validated the measurements of 72 volunteers^38^. And typically one or few experts verify the accuracy of voucher samples collected by large number of volunteers, e.g. for lady beetles^39^, or for invasive crabs^40^.

The units of sampling were plots of 100 m^2^. The plot sizes used were defined in the framework of the national habitat conservation status assessment, since this size accounted for the species area curve to achieve ecological consistency which formed the basis for the plot monitoring size. The number of plots examined is not equal to the number of participants. While the experts managed to collected data on multiple plots the non-experts collected data on only one plot each (either as an individual or as a team of two). We analyzed the data on the level of plots (between 30 and 60 plots per habitat type for non-experts and between 4 and 15 plots per habitat type for experts) but also cumulatively by pooling the information of all plots for a specific habitat type.

### Statistical analysis

We compared the conservation status assessment indicators recorded at each plot using permutational ANOVA. The indicators analyzed are the number of specific structures and functions per plot, the number of typical species per plot, and the number of pressures and threats per plot. As explanatory variable we used the status of the person recording the information with two classes (expert and non-experts), and the different habitat types (2120, 2270, 5210, and 9560). Firstly we compared all data using two way ANOVA with both habitat type and status as explanatory variables, and then we repeated the analysis for each habitat type separately using only status as explanatory variable. In all tests 5000 permutations were carried out using the package lmPerm in R^41^. Permutations were conducted on individuals, and were chosen because sample sizes were small, and also permutation test do not assume normal distributions and homogeneity of variances.

## Electronic supplementary material

Supplementary Information

## References

[1] Evans D (2012). Building the European Union’s Natura 2000 network. Nature conservation.

[2] Tsianou MA (2013). Identifying the criteria underlying the political decision for the prioritization of the Greek Natura 2000 conservation network. Biological conservation.

[3] Kallimanis AS (2015). Vegetation coverage change in the EU: patterns inside and outside Natura 2000 protected areas. Biodiversity and Conservation.

[4] Hernando A, Tejera R, Velázquez J, Núñez MV (2010). Quantitatively defining the conservation status of Natura 2000 forest habitats and improving management options for enhancing biodiversity. Biodiversity and Conservation.

[5] Evans, D. & Arvela, M. Assessment and reporting under Article 17 of the Habitats Directive – Explanatory Notes & Guidelines for the period 2007–2012. Final Draft, July 2011. *European Topic Centre on Biological Diversity* (2011).

[6] Corbane C (2015). Remote sensing for mapping natural habitats and their conservation status–New opportunities and challenges. International Journal of Applied Earth Observation and Geoinformation.

[7] Brambilla M (2009). GIS-models work well, but are not enough: Habitat preferences of *Lanius collurio* at multiple levels and conservation implications. Biological Conservation.

[8] Zlinszky A, Deák B, Kania A, Schroiff A, Pfeifer N (2015). Mapping Natura 2000 habitat conservation status in a pannonic salt steppe with airborne laser scanning. Remote Sensing.

[9] Tsiafouli MA (2013). Human activities in Natura 2000 sites: a highly diversified conservation network. Environmental management.

[10] Louette G (2011). Bridging the gap between the Natura 2000 regional conservation status and local conservation objectives. Journal for Nature Conservation.

[11] Mason F (2015). Monitoring of insects with public participation (MIPP; EU LIFE project 11 NAT/IT/000252): overview on a citizen science initiative and a monitoring programme (Insecta: Coleoptera; Lepidoptera; Orthoptera). Fragmenta entomologica.

[12] Conrad CC, Hilchey KG (2011). A review of citizen science and community-based environmental monitoring: issues and opportunities. Environmental monitoring and assessment.

[13] Dickinson JL, Zuckerberg B, Bonter DN (2010). Citizen science as an ecological research tool: challenges and benefits. Annual review of ecology, evolution and systematics.

[14] Schmeller DS (2008). European species and habitat monitoring: where are we now?. Biodiversity and Conservation.

[15] Pellissier V, Touroult J, Julliard R, Siblet JP, Jiguet F (2013). Assessing the Natura 2000 network with a common breeding birds survey. Animal Conservation.

[16] Sullivan BL (2014). The eBird enterprise: an integrated approach to development and application of citizen science. Biological Conservation.

[17] Matteson KC, Taron DJ, Minor ES (2012). Assessing citizen contributions to butterfly monitoring in two large cities. Conservation Biology.

[18] Zapponi, L. *et al*. Citizen science data as an efficient tool for mapping protected saproxylic beetles. *Biological Conservation* (2016).

[19] Hawthorne TL (2015). Mapping non-native invasive species and accessibility in an urban forest: A case study of participatory mapping and citizen science in Atlanta, Georgia. Applied Geography.

[20] Jordan RC, Brooks WR, Howe DV, Ehrenfeld JG (2012). Evaluating the performance of volunteers in mapping invasive plants in public conservation lands. Environmental management.

[21] Crall AW (2015). Citizen science contributes to our knowledge of invasive plant species distributions. Biological Invasions.

[22] Villaseñor E, Porter-Bolland L, Escobar F, Guariguata MR, Moreno-Casasola P (2016). Characteristics of participatory monitoring projects and their relationship to decision-making in biological resource management: a review. Biodiversity and Conservation.

[23] Gollan J, de Bruyn LL, Reid N, Wilkie L (2012). Can volunteers collect data that are comparable to professional scientists? A study of variables used in monitoring the outcomes of ecosystem rehabilitation. Environmental management.

[24] Snäll T, Kindvall O, Nilsson J, Pärt T (2011). Evaluating citizen-based presence data for bird monitoring. Biological conservation.

[25] Lukyanenko R, Parsons J, Wiersma YF (2016). Emerging problems of data quality in citizen science. Conservation Biology.

[26] Lewandowski E, Specht H (2015). Influence of volunteer and project characteristics on data quality of biological surveys. Conservation Biology.

[27] Fitzpatrick MC, Preisser EL, Ellison AM, Elkinton JS (2009). Observer bias and the detection of low‐density populations. Ecological Applications.

[28] Pescott OL (2015). Ecological monitoring with citizen science: the design and implementation of schemes for recording plants in Britain and Ireland. Biological Journal of the Linnean Society.

[29] Moyer‐Horner L, Smith MM, Belt J (2012). Citizen science and observer variability during American pika surveys. The Journal of Wildlife Management.

[30] Crall AW (2011). Assessing citizen science data quality: an invasive species case study. Conservation Letters.

[31] Anderson, D. R. et al. Field trials of line transect methods applied to estimation of desert tortoise abundance. The Journal of Wildlife Management, 583–597 (2001).

[32] Maciejewski L (2010). Méthodologie d’élaboration des listes d’ “espèces typiques” pour des habitats forestiers d’intérêt communautaire envue de l’évaluation de leurétat de conservation. Rapport SPN.

[33] European Commission. Interpretation Manual of European Union Habitats. EUR28. Brussels: European Commission (2013).

[34] Dimopoulos, P. *et al*. Identification and Interpretation Manual for the Forest Habitat Types of Greece. ISBN 978-960-9407-22-9. Katagramma editions, Kiato (2013).

[35] Delaney DG, Sperling CD, Adams S, Leung B (2008). Marine invasive species: validation of citizen science and implications for national monitoring networks. Biological Invasions.

[36] Galloway AW, Tudor MT, Haegen WMV (2006). The reliability of citizen science: a case study of Oregon white oak stand surveys. Wildlife Society Bulletin.

[37] Fuccillo KK, Crimmins TM, de Rivera CE, Elder TS (2015). Assessing accuracy in citizen science-based plant phenology monitoring. International journal of biometeorology.

[38] Finn PG, Udy NS, Baltais SJ, Price K, Coles L (2010). Assessing the quality of seagrass data collected by community volunteers in Moreton Bay Marine Park, Australia. Environmental Conservation.

[39] Gardiner MM (2012). Lessons from lady beetles: accuracy of monitoring data from US and UK citizen‐science programs. Frontiers in Ecology and the Environment.

[40] Delaney DG, Sperling CD, Adams CS, Leung B (2008). Marine invasive species: validation of citizen science and implications for national monitoring networks. Biological Invasions.

[41] Wheeler, B. & Torchiano, M. Package lmPerm Permutation Tests for Linear Models package version 2.1.0. (https://github.com/mtorchiano/lmPerm) (2016).

