# Supplementary material for: Quality of non-expert citizen science data collected for habitat type conservation status assessment in Natura 2000 protected areas
Source: Sci Rep. 2017 Aug 21;7:8873. doi: 10.1038/s41598-017-09316-9 (PMC5567195; doi:10.1038/s41598-017-09316-9)
Supplement: Supplementary file 1 — Supplementary Information [file 41598_2017_9316_MOESM1_ESM.pdf]

Quality of non-expert citizen science data collected for habitat type conservation status assessment in Natura 2000 protected areas.

Kallimanis A.S. [1,2], Panitsa M.[2,3], Dimopoulos P.[2,3]

[1.] Department of Ecology, Aristotle University, Thessaloniki, Greece

[2.] Department of Environmental and Natural Resources Management, University of Patras, Agrinio, Greece.

[3.]Faculty of Biology, Division of Plant Biology, University of Patras, Patras, Greece

Supplementary material

Protocols for collecting field data towards the conservation status assessment of habitat types:

2120 "Shifting dunes along the shoreline with *Ammophila arenaria* (white dunes)"

2270 "Wooded dunes with *Pinus pinea* and/or *Pinus pinaster*"

5210 "Arborescent matorral with *Juniperus* spp."

9560 "Endemic forests with *Juniperus* spp."

2120 "Shifting dunes along the shoreline with *Ammophila arenaria* (white dunes)" – all clusters

**Evaluator:**			**Site code:**		
**Plot code (ddmmyy#nbr):**			**Existing relevé nr:**		
**Date of assessment:**					
**coords**	**centre**	**LON:**	**bottom right**	**LON:**	
		**LAT:**		**LAT:**	
**Locality:**					
**Plot size (m^2) (sugg. = 16 m^2):**			**Area assessed (m^2) (sugg. 100 m^2):**		
**Exposition (°):**			**Altitude (m):**		
**Inclination (°):**			**Relief: cliff / slope / plain / depression/ravine**		
**Geological substratum:**			**Soil type: sandy - silt - loam**		
**picts**	**CENTRE**	**id:**	**azimuth:**		
	**PANOR**	**id:**	**azimuth:**	**LON:**	
				**LAT:**	
**Adjacent vegetation (habitat) type(s):**			**Substratum with significant disturbances (e.g. erosion, trampling)**	**YES** / **NO**	
**Invasive/Ruderal species (incl. abundance):**					
**width of sand dunes:**			**distance from sea:**		
**Other:**					

	Cover (%)				
	**0-5**	**5-25**	**25-50**	**50-75**	**>75**
**boulders (>20 cm)**					
**stones (2-20 cm)**					
**gravel (2mm – 2cm)**					
**fine soil**					
**litter**					
**moss**					

Layer	Cover %	Height (m)
**Tree (>2m)**		
**Shrub (0.5-2 m)**		
**Herb (<0.5m)**		

Specific Structure and Functions					
☐	Sparse vegetation cover ≥ 20%	☐	Discrete spatial succession pattern	☐	Substrate with no significant disturbances
☐	Communities mainly consisted of rhizomatous geophytes and hemicryptophytes favouring sand dune formation by trapping blowing sand	☐	Vegetation of yellow, very permeable and humus poor mobile sand dunes	☐	Dunes usually more than 1 m high
☐	Dunes landwards of the *Agropyretum juncei* (2110)	☐		☐	

Prospects of Structure and Functions			
**Future Trend**			
☐ FV<br>No P or T of high importance and up to 1 of medium importance or positive impacts balance higher number or importance of P or T	☐ U1<br>Up to 3 P or T of medium importance or positive impacts balance higher number or importance of P or T	☐ U2<br>At least 1 T or P of high importance and/or more than 3 P or T of medium importance without positive impacts being able to balance them	☐ XX<br>Not able to assess P or T
**Future status**			
☐ FV<br>Struct. & funct. are expected to be in FV status in more than 75% of the studied locality	☐ U1<br>Struct. & funct. are expected to be in FV stat in 50-75% of the studied locality and not more than 25% in U2 status	☐ U2<br>Struct. & funct. are expected to be in FV status in less than 50% of the studied locality or more than 25% in U2 status	☐ XX:<br>Not able to asses future conservation status in > 50% of the studied locality
**Restoration possibility**			
☐ easy	☐ possible with an average effort	☐ difficult or impossible	☐ unable to assess

Positive impacts (management actions, policy changes etc). Description and importance.

Description	Importance

Notes:

2120 "Shifting dunes along the shoreline with *Ammophila arenaria* (white dunes)" – all clusters

Typical species							
**Species**	**Cover**		**Vit.**	**Species**	**Cover**		**Vit.**
	**plot**	**area**			**plot**	**area**	
Ammophila arenaria				*Medicago littoralis*			
Anthemis tomentosa				*Medicago marina*			
Artemisia campestris				*Nigella arvensis*			
Artemisia maritima agg.				*Ononis natrix*			
Atriplex tatarica				*Otanthus maritimus*			
Bromus tectorum				*Pancratium maritimum*			
Cakile maritima				*Parapholis incurva*			
Centaurea aegialophila				*Phragmites australis*			
Centaurea grisebachii				*Plantago arenaria*			
Centaurea spinosa				*Plantago coronopus*			
Chondrilla juncea				*Plantago lagopus*			
Cichorium intybus				*Plantago weldenii*			
Crepis foetida				*Polygonum maritimum*			
Cutandia maritima				*Pseudorlaya pumila*			
Cynodon dactylon				*Salsola kali*			
Cyperus capitatus				*Silene ammophila*			
Echium arenarium				*Silene colorata*			
Elymus farctus				*Silene euxina*			
Elymus rechingeri				*Silene niceensis*			
Erodium laciniatum				*Silene sedoides*			
Eryngium maritimum				*Silene subconica*			
Euphorbia paralias				*Silene subconica*			
Euphorbia peplis				*Silene succulenta*			
Euphorbia terracina				*Sporobolus pungens*			
Hedypnois cretica				*Tamarix hampeana*			
Jasione heldreichii				*Triplachne nitens*			
Leymus racemosus				*Verbascum pinnatifidum*			
Limonium gmelinii				*Vulpia fasciculata*			
Lotus halophilus				*Xanthium strumarium*			
Matthiola tricuspidata				*Zygophyllum album*			

Pressures (P) and Threats (T) for the habitat type			
**Code**	**Description**	**P, T, T or PT**	**Importance**

2270 “Wooded dunes with *Pinus pinea* and/or *Pinus pinaster*” - all clusters

**Evaluator:**			**Site code:**		
**Plot code (ddmmyy#nbr):**			**Existing relevé nr:**		
**Date of assessment:**					
**coords**	**centre**	**LON:**	**bottom right**	**LON:**	
		**LAT:**		**LAT:**	
**Locality:**					
**Plot size (m^2) (sugg. = 16 m^2):**			**Area assessed (m^2) (sugg.=100 m^2):**		
**Exposition (^{o}):**			**Altitude (m):**		
**Inclination (^{o}):**			**Relief:** cliff / slope / plain / depression/ravine		
**Geological substratum:**			**Soil type:** sandy - silt - loam		
**picts**	**CENTRE**	**id:**	**azimuth:**		
	**PANOR**	**id:**	**azimuth:**	**LON:**	
				**LAT:**	
Adjacent vegetation (habitat) type(s):			Substratum with significant disturbances (e.g. erosion, trampling)	**YES**<br>**NO**	
Invasive/Ruderal species (incl. abundance):					
width of sand dunes:			distance from sea:		
Other:					

	Cover (%)				
	**0-5**	**5-25**	**25-50**	**50-75**	**>75**
**boulders (>20 cm)**					
**stones (2-20 cm)**					
**gravel (2mm – 2cm)**					
**fine soil**					
**litter**					
**moss**					

**Layer**	**Cover %**	**Height (m)**
**Tree (>2m)**		
**Shrub (0.5-2 m)**		
**Herb (<0.5m)**		

Ειδικές Δομές και Λειτουργίες

☐	*Pinus pinea* tree density medium to high in pure stands	☐	*Pinus pinea* species natural regeneration and diversity of species age classes	☐	Substrate with no significant disturbances (e.g. trampling)
☐	Communities with no significant disturbances characterized by typical floristic structure	☐	Absence of gradual replacement of the *Pinus pinea* trees by *Pinus halepensis* in the middlestorey	☐	Absence of gradual replacement of the *Pinus pinea* trees by *Pinus halepensis* in the understorey
☐	Discrete *Pinus pinea* stands covering medium sized to extensive areas (500 m2 <x> ha)	☐	Stand stratified (tree, shrub, herb layers present)	☐	Natural *Pinus pinea* stands with more than one third of the individuals at reproductive age
☐		☐		☐	

**Prospects of Structure and Functions**			
**Future Trend**			
☐ FV<br>No P or T of high importance and up to 1 of medium importance or positive impacts balance higher number or importance of P or T	☐ U1<br>Up to 3 P or T of medium importance or positive impacts balance higher number or importance of P or T	☐ U2<br>At least 1 T or P of high importance and/or more than 3 P or T of medium importance without positive impacts being able to balance them	☐ XX<br>Not able to assess P or T
**Future status**			
☐ FV<br>Struct. & funct. are expected to be in FV status in more than 75% of the studied locality	☐ U1<br>Struct. & funct. are expected to be in FV stat in 50-75% of the studied locality and not more than 25% in U2 status	☐ U2<br>Struct. & funct. are expected to be in FV status in less than 50% of the studied locality or more than 25% in U2 status	☐ XX:<br>Not able to asses future conservation status in > 50% of the studied locality
**Restoration possibility**			
☐ easy	☐ possible with an average effort	☐ difficult or impossible	☐ unable to assess

**Positive impacts** (management actions, policy changes etc). Description and importance.	
Description	Importance

Notes:

2270 “Wooded dunes with *Pinus pinea* and/or *Pinus pinaster*” - all clusters

typical species							
**species**	**cover**		**vit.**	**species**	**cover**		**vit.**
	**plot**	**area**			**plot**	**area**	
Aegilops species				*Muscari neglectum*			
Aeluropus littoralis				*Myrtus communis*			
Anemone pavonina				*Ononis species*			
Arbutus unedo				*Ornithopus compressus*			
Arum maculatum				*Pinus halepensis*			
Asparagus acutifolius				*Pinus pinea*			
Brachypodium distachyon				*Piptatherum miliaceum*			
Briza maxima				*Pistacia lentiscus*			
Bupleurum species				*Prasium majus*			
Carlina corymbosa				*Quercus coccifera*			
Centranthus calcitrapae				*Quercus ilex*			
Cistus monspeliensis				*Rhamnus alaternus*			
Cistus salviifolius				*Rubia peregrina*			
Cynosurus echinatus				*Rubus ulmifolius*			
Ephedra foeminea				*Rumex species*			
Erica arborea				*Ruscus aculeatus*			
Euphorbia exigua				*Salvia verbenaca*			
Euphorbia peplus				*Scaligeria napiformis*			
Geranium robertianum				*Senecio lividus*			
Hypochaeris achyrophorus				*Silene colorata*			
Juniperus phoenicea				*Silene gallica*			
Lagurus ovatus				*Smilax aspera*			
Lathyrus cicera				*Torilis nodosa*			
Leontodon tuberosus				*Trifolium angustifolium*			
Linaria pelisseriana				*Vicia lathyroides*			
Linum species				*Vicia species*			
Medicago littoralis				*Vicia villosa*			

Pressures (P) and Threats (T) for the habitat type			
**Code**	**Description**	**P, T, T or PT**	**Importance**

5210 ''Arborescent matorral with *Juniperus* spp.'' – all clusters

**Evaluator:**			**Site code:**		
**Plot code (ddmmyy#nbr):**			**Existing relevé nr:**		
**Date of assessment:**					
**coords**	**centre**	**LON:**	**bottom right**	**LON:**	
		**LAT:**		**LAT:**	
**Locality:**					
**Plot size (m^2) (sugg. = 50 m^2):**			**Area assessed (m^2) (sugg. = 500 m^2):**		
**Exposition (°):**			**Altitude (m):**		
**Inclination (°):**			**Relief:** cliff / slope / plain / depression/ravine		
**Geological substratum:**			**Soil type:** sandy - silt - loam		

**picts**	**CENTRE**	**id:**	**azimuth:**	
	**PANOR**	**id:**	**azimuth:**	**LON:**
				**LAT:**

Adjacent vegetation (habitat) type(s):		Substratum with significant disturbances (e.g. erosion, trampling)	**YES** **NO**
Invasive/Ruderal species (incl. abundance):			
Other:			

	Cover (%)				
	**0-5**	**5-25**	**25-50**	**50-75**	**>75**
**boulders (>20 cm)**					
**stones (2-20 cm)**					
**gravel (2mm – 2cm)**					
**fine soil**					
**litter**					
**moss**					

**Layer**	**Cover %**	**Height (m)**
**Tree (>2m)**		
**Shrub (0.5-2 m)**		
**Herb (<0.5m)**		

**Specific Structure and Functions**					
☐	Upper storey dominated by high evergreen shrubs	☐	Understory dominated by phrygana and/or therophytes	☐	Absence of illegal logging
☐	Presence/practice of normal (regular) grazing	☐	Absence of dense road network or infrastructures	☐	Absence of evidence(s) of primary or secondary succession
☐	Absence of indications of significant/important erosion (e.g. crevasse-like erosion)	☐	Rich in important plant species community	☐	Presence of juniper seedlings

Notes:

**Prospects of Structure and Functions**			
**Future Trend**			
☐ FV<br>No P or T of high importance and up to 1 of medium importance or positive impacts balance higher number or importance of P or T	☐ U1<br>Up to 3 P or T of medium importance or positive impacts balance higher number or importance of P or T	☐ U2<br>At least 1 T or P of high importance and/or more than 3 P or T of medium importance without positive impacts being able to balance them	☐ XX<br>Not able to assess P or T
**Future status**			
☐ FV<br>Struct. & funct. are expected to be in FV status in more than 75% of the studied locality	☐ U1<br>Struct. & funct. are expected to be in FV stat in 50-75% of the studied locality and not more than 25% in U2 status	☐ U2<br>Struct. & funct. are expected to be in FV status in less than 50% of the studied locality or more than 25% in U2 status	☐ XX:<br>Not able to asses future conservation status in > 50% of the studied locality
**Restoration possibility**			
☐ easy	☐ possible with an average effort	☐ difficult or impossible	☐ unable to assess

Positive impacts (management actions, policy changes etc). Description and importance.

Description	Importance

5210 ''Arborescent matorral with *Juniperus* spp.'' – all clusters

Typical species							
**Species**	**Cover**		**Vit.**	**Species**	**Cover**		**Vit.**
	**plot**	**area**			**plot**	**area**	
Acinos alpinus				*Allium subhirsutum*			
Aetheorhiza bulbosa				*Anagallis arvensis*			
Arisarum vulgare				*Asphodelus ramosus*			
Asterolinon linum-stellatum				*Astragalus creticus*			
Asperula pulvinaris				*Atractylis cancellata*			
Avena barbata				*Brachypodium distachyon*			
Brachypodium pinnatum				*Brachypodium retusum*			
Briza maxima				*Bupleurum gaudianum*			
Bupleurum flavum				*Bupleurum gracile*			
Calendula arvensis				*Calicotome villosa*			
Carex liparocarpos				*Catapodium rigidum*			
Centaurea laconica				*Centaurea raphanina*			
Cerastium candidissimum				*Cirsium candelabrum*			
Coridothymus capitatus				*Crataegus pycnoloba*			
Crucianella latifolia				*Cynoglossum creticum*			
Cynara cornigera				*Daphne oleoides*			
Eryngium amethystinum				*Euphorbia acanthothamnos*			
Euphorbia exigua				*Euphorbia myrsinites*			
Fumana arabica				*Fumana procumbens*			
Gladiolus illyricus				*Globularia bisnagarica*			
Helianthemum salicifolium				*Hippocrepis unisiliquosa*			
Hymenonema graecum				*Hypochaeris achyrophorus*			
Juniperus oxycedrus				*Lagoecia cuminoides*			
Leontodon tuberosus				*Limonium antipaxorum*			
Linum strictum				*Linum trigynum*			
Malcolmia maritima				*Melica ciliata*			
Micromeria graeca				*Micromeria nervosa*			
Minuartia attica				*Myrtus communis*			
Nepeta camphorata				*Nigella arvensis*			
Olea europaea				*Ononis reclinata*			
Paronychia albanica				*Paronychia macrosepala*			
Paronychia rechingeri				*Periploca angustifolia*			
Phagnalon graecum				*Pistacia lentiscus*			
Piptatherum miliaceum				*Plantago bellardii*			
Pisum sativum				*Prasium majus*			
Potentilla arcadiensis				*Prunus cocomilia*			
Ptilostemon afer				*Rhamnus lycioides*			
Rosa heckeliana				*Rostraria cristata*			
Selaginella denticulata				*Sideritis raeseri*			
Silene radicosa				*Smilax aspera*			
Stipa capensis				*Stipa capillata*			
Stipa pennata				*Teucrium brevifolium*			
Teucrium capitatum				*Thymus longicaulis*			
Thymus rechingeri				*Thymus sipyleus*			
Trifolium campestre				*Trigonella coerulescens*			
Tuberaria guttata				*Urginea maritima*			
Valantia hispida				*Valantia muralis*			
Juniperus macrocarpa							
Juniperus phoenicea							
Juniperus excelsa				*Juniperus foetidissima*			

Pressures (P) and Threats (T) for the habitat type			
**Code**	**Description**	**P, T, T or PT**	**Importance**

9560 "Endemic forests with *Juniperus spp.*" – all clusters

**Evaluator:**			**Site code:**	
**Plot code (ddmmyy#nbr):**			**Existing relevé nr:**	
**Date of assessment:**				
**coords**	**centre**	**LON:** / **LAT:**	**bottom right**	**LON:** / **LAT:**
**Locality:**				
**Plot size (m^2) (sugg. = 200 m^2):**			**Area assessed (m^2) (sugg. = 500 m^2):**	
**Exposition (^{o}):**			**Altitude (m):**	
**Inclination (^{o}):**			**Relief:** cliff / slope / plain / depression/ravine	
**Geological substratum:**			**Soil type:** sandy - silt - loam	

**picts**	**CENTRE**	**id:**	**azimuth:**	
	**PANOR**	**id:**	**azimuth:**	**LON:** / **LAT:**

Adjacent vegetation (habitat) type(s):		Substratum with significant disturbances (e.g. erosion, trampling)	**YES** / **NO**
Invasive/Ruderal species (incl. abundance):			
Other:			

	Cover (%)				
	**0-5**	**5-25**	**25-50**	**50-75**	**>75**
**boulders (>20 cm)**					
**stones (2-20 cm)**					
**gravel (2mm – 2cm)**					
**fine soil**					
**litter**					
**moss**					

**Layer**	**Cover %**	**Height (m)**
**Tree (>2m)**		
**Shrub (0.5-2 m)**		
**Herb (<0.5m)**		

Specific Structure and Functions

☐	Cover of shrub and tree layers of *Juniperus* species higher than 30%	☐	Soil covered with litter for more than 20% of the plot area	☐	At least 30% of *Juniperus* species individuals with tree like form
☐	Adequate regeneration of *Juniperus* species both in the herb and in the shrub layers	☐	Absence or low cover (<5%) of ruderal and/or invasive species	☐	Stand without signs of significant disturbance (e.g. from logging, grazing, fires, natural causes such as windfalls)
☐	Diversity of *Juniperus* species age classes	☐	No signs of erosion or only sheet (surface) erosion (furrows with depth <30 cm) present in less than 20% of the area	☐	Absence of planted species (e.g. from reforestation)
☐	Cover of forest herb species (shade tolerant species) >25%	☐		☐	

Prospects of Structure and Functions

**Future Trend**			
☐ FV<br>No P or T of high importance and up to 1 of medium importance or positive impacts balance higher number or importance of P or T	☐ U1<br>Up to 3 P or T of medium importance or positive impacts balance higher number or importance of P or T	☐ U2<br>At least 1 T or P of high importance and/or more than 3 P or T of medium importance without positive impacts being able to balance them	☐ XX<br>Not able to assess P or T
**Future status**			
☐ FV<br>Struct. & funct. are expected to be in FV status in more than 75% of the studied locality	☐ U1<br>Struct. & funct. are expected to be in FV stat in 50-75% of the studied locality and not more than 25% in U2 status	☐ U2<br>Struct. & funct. are expected to be in FV status in less than 50% of the studied locality or more than 25% in U2 status	☐ XX:<br>Not able to asses future conservation status in > 50% of the studied locality
**Restoration possibility**			
☐ easy	☐ possible with an average effort	☐ difficult or impossible	☐ unable to assess

Positive impacts (management actions, policy changes etc). Description and importance.

Description	Importance

Notes:

9560 "Endemic forests with *Juniperus spp.*" – all clusters

Typical species							
**Species**	**Cover**		**Vit.**	**Species**	**Cover**		**Vit.**
	**plot**	**area**			**plot**	**area**	
Abies cephalonica				*Hippocrepis emerus*			
Acer monspessulanum				*Hypericum barbatum*			
Acinos alpinus				*Juniperus drupacea*			
Aethionema saxatile				*Juniperus excelsa*			
Allium flavum				*Juniperus foetidissima*			
Alyssum montanum				*Juniperus oxycedrus*			
Arabis glabra				*Lamium garganicum*			
Armeria canescens				*Leontodon crispus*			
Asparagus acutifolius				*Lonicera etrusca*			
Asperula aristata				*Malcolmia graeca*			
Astragalus angustifolius				*Marrubium velutinum*			
Astragalus creticus				*Melica ciliata*			
Asyneuma limonifolium				*Melica uniflora*			
Aubrieta deltoidea				*Morina persica*			
Brachypodium pinnatum				*Onobrychis alba*			
Brachypodium retusum				*Phillyrea latifolia*			
Buxus sempervirens				*Prunus cocomilia*			
Campanula spatulata				*Prunus spinosa*			
Carex distachya				*Pteridium aquilinum*			
Carex flacca				*Pterocephalus perennis*			
Carex illegitima				*Pyrus spinosa*			
Carpinus orientalis				*Quercus coccifera*			
Cerastium candidissimum				*Ranunculus brevifolius*			
Crepis fraasii				*Rhamnus saxatilis*			
Daphne oleoides				*Ribes uva-crispa*			
Eryngium amethystinum				*Rubia peregrina*			
Erysimum parnassi				*Scabiosa columbaria*			
Erysimum pectinatum				*Scrophularia canina*			
Euphorbia apios				*Scutellaria rupestris*			
Festuca jeanpertii				*Sesleria vaginalis*			
Festuca polita				*Silene italica*			
Fritillaria mutabilis				*Spartium junceum*			
Galium peloponnesiacum				*Stipa bromoides*			
Galium thymifolium				*Teucrium chamaedrys*			
Genista parnassica				*Trifolium medium*			
Geocaryum capillifolium				*Trisetum flavescens*			
Geranium lucidum				*Viola alba*			
Helictotrichon convolutum							

Pressures (P) and Threats (T) for the habitat type			
**Code**	**Description**	**P, T, T or PT**	**Importance**
